# Do Children Think it is Important to Predict Learning and Behaviour Problems, and Do They Think Genetic Screening Has a Role to Play in This?

**DOI:** 10.1007/s10803-023-05966-z

**Published:** 2023-04-06

**Authors:** Diana Fields, Kathryn Asbury

**Affiliations:** Psychology in Education Research Centre, Department of Education, University of York, Heslington, United Kingdom

**Keywords:** polygenic scores, additional needs, learning difficulties, behaviour problems, genetics, neurodivergence, children’s views

## Abstract

This study explores how capable young children are of thinking about a potential future that uses DNA screening to assess an individual’s likelihood of experiencing learning or behaviour difficulties. Puppets and a scenario-based approach were used to ask children aged 4–10 (*n = 165*) whether they thought DNA screening might be helpful or harmful. A content analysis derived six categories: (1) ‘Worried about being – and being seen as – different’; (2) ‘Beliefs about the origins of learning and behaviour’; (3) ‘Testing is harmful’; (4) ‘Testing could help’; (5) ‘How soon is too soon for testing?’; and (6) ‘What’s the point?’. Findings indicate young children, as key stakeholders, can make useful contributions to public debate in this important and controversial area.

Adults, on average, have low levels of genetic literacy and this appears to have consequences for their beliefs about genetic influences on human behaviour and their perceptions of DNA screening (Chapman et al., 2019; Rew et al., 2010). Genetic literacy has been defined as “sufficient knowledge and understanding of genetic principles to make decisions that sustain personal well-being and effective participation in social decisions on genetic issues” (Little & Gunter, 2021, p. 2). Chapman et al. (2019) suggest that understanding genetic information “is becoming increasingly crucial for all aspects of our lives” including job prospects and educational attainment (Chapman et al., 2019, p. 73). However, it is important to acknowledge that not everybody accepts that applying findings from genomic research to outcomes such as educational attainment – and their implications for social policy - is appropriate or wise (Coop & Przeworski, 2022).

Improving public understanding of genetics has been found to enhance health behaviours and to reduce adolescents’ false beliefs and biologically deterministic thinking in relation to social identity, culture, and race (Donovan et al., 2021). By contrast, little is known about younger children’s views or understanding regarding genetic influences on individual differences in learning and behaviour (including neurodivergence and developmental disorders), or about a possible future in which DNA screening for such traits or diagnoses could potentially be used (Asbury et al., 2021). This is problematic because any decisions made about genetic screening for probability of experiencing learning and behaviour difficulties will directly affect children. Responsible research therefore demands that their voices should be heard in discussions about the future of genetic screening (Carrier & Gartzlaff, 2020).

Individual differences in educationally relevant traits such as cognitive ability, attention and self-regulation are partly explained by genetic differences between individuals (Polderman et al., 2015). International teams have begun to identify genetic variants of small effect that correlate with behavioural traits, such as years of education and cognitive ability, and to combine them in genomewide polygenic scores (GPS) that explain small to moderate proportions of variance (Okbay et al., 2022). Commercial companies have begun to use GPSs for screening purposes, albeit prematurely (Munday & Savulescu, 2021; Turley et al., 2021; Lázaro-Muñoz et al., 2021). Services include products that screen DNA for information on “ancestry and predisposition to various diseases and health issues” (Saha et al., 2020, p. 120).

Some researchers have argued that there is potential for GPSs to be used within education, in conjunction with other assessment tools, to identify the probability of needing additional support e.g. as a result of learning difficulties (Asbury & Plomin, 2013; Shero et al. 2021) although this remains contentious (Asbury et al., 2021). It seems highly likely that GPSs will become increasingly available, but there are risks associated with this when society is not sufficiently prepared to understand the implications of using them, including their limits, and to put the necessary safeguards in place (Plomin & von Stumm, 2018). It is important to note in this context that what we consider to be difficulties or problems in relation to learning and behaviour is governed, to some extent, by cultural and social factors (Becker, 1963). Because being labelled as having a learning difficulty or behaviour problems has practical implications for children, and for their parents and teachers, this is something we need to consider.

It is important to know what children think about genetics because they are key stakeholders in discussions about DNA-based prediction of learning difficulties and behaviour problems. It is also important, in this context, to understand children’s views of those they perceive as different to themselves, including those who are neurodivergent, which may or may not be associated with learning difficulties or behaviour problems but is likely to manifest as differing from the neurotypical norm in some way. This can enhance current understanding of the risks of stigma, self-stigma and detrimental expectancy effects that may be associated with DNA screening for the likelihood of being labelled as having learning difficulties or behaviour problems (Shifrer, 2013).

To date, little is known about how children view divergent learning and behaviour profiles (Beckett, 2014; Cairns & McClatchey, 2013). The same is also true for research into how young children develop genetic literacy (Meyer et al., 2020). However, there is a relevant body of literature regarding how and when children form essentialist and deterministic perspectives about difference, sameness, and inclusion. The available literature suggests that neurotypical children tend to view those with learning difficulties and behaviour problems, which may include some neurodivergent children, as not being able to do anything and to categorize others as ‘normal’ (Beckett, 2014). These categorisations have also been found to be perpetuated through the media (Samsel & Perepa, 2013). Children appear to make a distinction between’ kids like me’ and ‘kids like them’, and to show a clear preference for homophily (Schwab, 2019, p. 9).

We also know that children develop essentialist thinking, the belief that categories share certain attributes, that cannot be seen (e.g. tigers are fierce) very early in childhood, from the age of 2, (Gelman, 2004). They begin to search for hidden, nonobvious, and fixed attributes such as fierceness in a wide range of categories (Gelman, 2004; Gelman et al., 1998; Heyman & Gelman, 2000). By the age of 4–5 children perceive category membership (e.g. being a tiger) as stable and causal (e.g. tigers are always fierce) (Gelman, 2004). Children’s thinking at 2 years shows that they are able to make simple inferences from new information about a category and to try to generalise that fact to other category members. By the age of 4–5 children’s thinking becomes more sophisticated and stable as they decide whether or not they generalise from individual observations to whole categories in particular cases (Gelman, 2004).

Baldwin et al. (1993) have argued that such psychological essentialism happens even earlier than the age of 2, showing that children as young as 9 months, after being shown objects once, can make straightforward interpretations about the hidden makeup of things (Baldwin et al., 1993). Gelman et al., (1998) claim that children are not taught how to categorize by their parents, making the argument that this behaviour is innate, but they acknowledge that the language used by parents when describing objects or animals, and indeed the language not used, may support children’s assumptions about certain categories (e.g. fierce tigers in pretend play interactions). The development of essentialist thinking could be seen as important to the development of children’s social attitudes towards those who look, learn, or behave differently from themselves as this too involves defining or acknowledging categories and their characteristics (Prentice & Miller, 2007).

Psychological essentialism has commonalities with other psychological biases such as correspondence bias (Gilbert & Malone, 1995), beliefs about the self (Dweck and Leggett 1998) and that social groups share coherence and unity (Campbell, 1958). They all share the perspective that we tend to see others as having unseen and permanent characteristics (Dar-Nimrod & Heine, 2011). Genetic essentialism refers to the assumption that human traits and attributes are established from a genetic make-up which is immovable and deterministic (Dar-Nimrod & Heine, 2011). One risk of perceiving genetic effects to be immutable, combined with psychological essentialism, is that this perspective may lead to particular groups being viewed as permanently similar or distinct, with members of that group (e.g. children who present with learning or behaviour difficulties), like tigers, having the same “genetic essence” (Dar-Nimrod & Heine, 2011, p. 4).

It seems that the capacity for category formation stabilises by age 5. This knowledge suggests a window of opportunity – in the earliest years of schooling - for opening up constructive discussions about how and why children differ from each other in order to work towards changing incorrect but stable beliefs and developing positive and accepting relationships. This is worth doing early because we also know that children have the capacity to question and change their perceptions through interventions that discuss and challenge perceived differences (Gus, 2000; Beckett, 2014; McGill, 2019). This indicates the potential importance of educating young children about individual differences in learning and behaviour and their aetiology in age-appropriate ways from the start of school (Cairns & McClatchey, 2013; Black-Hawkins et al., 2021).

By eliciting and amplifying children’s voices on these important topics – behavioural diversity and genetic prediction - educational practitioners can provide a vital platform for children to be heard and mediate between educational policy and children’s perspectives (Wall, 2017; Murray, 2019). The use of innovative methodologies such as Philosophy for Children (P4C) and age-appropriate data collection approaches (Lees et al., 2017) including resources such as puppets (Dunst, 2014) – as used in the current study - could be useful tools in the development of this important platform.

## Child Friendly Methods of Data Collection

Matthew Lipman originally established philosophy for children (P4C) in 1970. He was motivated by the work of psychologists such as Vygotsky and the philosopher Socrates and this is strongly reflected in the P4C approach (Daniel et al. 2011).

During a P4C session the teacher acts as a facilitator, initially choosing the stimulus but letting the children choose where the inquiry will lead. The basic procedure includes a warm-up activity to practice P4C’s 4 C skills of being Caring, Creative, Cooperative and Critical. Next the stimulus is presented. In the case of the P4C session underpinning the current study, that was the introduction of a video called ‘Alexandra’s Story, Same but Different’ (see below for further details). After that, children are asked to think about what they have just heard and seen. The teacher asks the children to respond to an opening question. They then facilitate discussion by encouraging children to agree or disagree with others’ perspectives and to build on each other’s points or explain any disagreement. At the end, children are asked to reflect on the session and on whether the discussion has affected their viewpoints.

The P4C goal is to enable children to listen to the views of other children and to support them to give their views in a logical, reflective manner in collaboration with others. Anderson (2016) explains how important P4C is for confronting beliefs with regard to understanding and ideas, particularly with regard to others in the P4C group and beyond. P4C is designed to promote a desire to know, develop analytical thinking skills and cultivate reasoning and discussion with a view to increasing children’s knowledge and understanding. P4C therefore represents a novel and useful way to understand the development of children’s thinking about difference and its, partially genetic, aetiology.

There are also potential benefits to using puppets as a pedagogical tool for intervention, which arguably have not been fully taken advantage of by teachers and schools (Kröger & Nupponen, 2019). Puppets have the potential to change young children’s attitudes and understanding of divergence, particularly when used in conjunction with other interventions which engage children in collaborative exercises, such as P4C (Dunst, 2014). The current study employed puppets to build playful and relaxed relationships with participants (Kröger & Nupponen, 2019) and to offer them an opportunity to freely and appropriately express themselves (Korosec, 2013) about their perspectives on children who learn or behave differently to themselves, and the potential of screening for learning and behaviour problems, and potentially forms of neurodivergence that may be associated with learning and behaviour problems, from infancy.

To our knowledge, this is the first study to ask children as young as age 4 what they think about a future in which adults could potentially screen infants for their likelihood of experiencing learning or behaviour difficulties, or for being different in some other way. The study was designed using child-friendly methods (Aldridge, 2017) to elicit even very young children’s understanding of the aetiology of learning and behaviour difficulties, and their perceptions about screening for the probability of experiencing them at birth. It was designed on the understanding that responsible research and innovation requires us to listen to children’s views when planning the future use of DNA-based data so that we can (a) establish principles to avoid harm, (b) put appropriate regulation in place that aligns with children’s perspectives (United Nations Convention on the Rights of the Child, 1989) and c) hear and act upon children’s views as key stakeholders in these discussions (Bradwell, 2019). The research questions were:

1. How do children perceive life with learning or behaviour difficulties?
2. Do young children believe that genetic screening for learning and behaviour difficulties would be helpful or harmful?

## Methods

### Participants

Participants were 165 children recruited from two primary schools in the north of England (*n* = 63 Early Years Foundation Stage (EYFS), age 4–5, 55 Year 2, age 6–7 and 47 Year 5, age 9–10)., School 1 participants were recruited in the Autumn term 2019 (*n* = 111) and School 2 participants in Spring term 2020 (*n* = 54). All children, regardless of special educational needs and disabilities (SENDs), were invited to participate in the study. There were 39 children who had been diagnosed with SENDs, including learning difficulties and behaviour problems, who took part. In School 1 there were 6 such children from EYFS, 6 from Year 2 and 10 from Year 5 (totalling 22 children). In school 2, 3 children with SENDs participated from EYFS, 4 from Year 2 and 10 from Year 5 (totalling 17 children).

Both schools were experienced in the use of P4C as this was a prerequisite for participating in the study.

### Ethical Considerations

The study adhered to the authors’ University Code of Practice and principles for good ethical governance. Ethical approval was granted by the Ethics Committee of the authors’ department.

Chervin and Kyle (1993) suggest that using collaborative inquiry with school pupils as a research method is ethically, academically, and politically plausible, even though children have sometimes been viewed as deficient “in their reasoning capacity and competence” (p. 29). Chervin and Kyle (1993) argued that, in many collaborative studies, children continued to be ‘worked on’, rather than ‘with’ as “research partners” (p. 30). We took several steps to avoid this.

The British Educational Research Association, BERA, (2018) ethics guidance suggests that support for children, when asking them to assent to research participation, is needed. Gaining assent from children needs ‘time and constant effort’ (Cocks, 2006, p. 257) on behalf of the researcher. Young children are able to make informed decisions, if they understand the context and it is meaningful to them (Coyne, 2010).

Support for child assent was particularly important in the current study because we were exploring a sensitive topic. It was also important to consider data-collection challenges such as adult-child power imbalances when interviewing children (Urbina-Garcia, 2019) and the complexity of designing and implementing self-report measures (Coombes et al., 2021). The aim was to empower children by giving them an appropriate opportunity to express their viewpoints and take them seriously (Urbina-Garcia, 2019). Working with children (Dockett & Perry, 2011), listening to, and acting upon their perspectives was central to this study (Convention on the Rights of the Child, 1989; Dockett & Perry, 2011).

Gaining children’s assent was a particularly important factor, and was achieved in the following ways:

The invitation letter sent to parents, via the school, asked parents or carers to discuss with their child what the research was about and what it would mean for them if they took part. Parents were asked to opt their child into the research rather than taking an opt-out approach, after talking with their child about what taking part in the study would mean for them.

Parents and children, through the invitation letter, were made aware that they could withdraw at any time, without reason, and that there would be no penalty i.e. that children would not be in trouble for saying they didn’t want to continue. This meant that from the start of the intervention children were asked if they were happy to participate at each stage (pre, intervention, post, post delayed testing). This also meant observing children’s non-verbal behaviour for signs that they didn’t want to continue. The first author, as a former teacher of EYFS and the primary age group, had a very good understanding of children’s behaviour within a school context. While engaged in data collection, the first author was aware that children’s assent may change at any given moment and was particularly aware of the three main types of dissent ‘normative, unnoticeable and playful’ (Kirby, 2020), which could be displayed through non-verbal body language, and to act ethically by ensuring that the children were able to continue to make an informed choice about participation (Whittington, 2019).

**Fig. 1:**
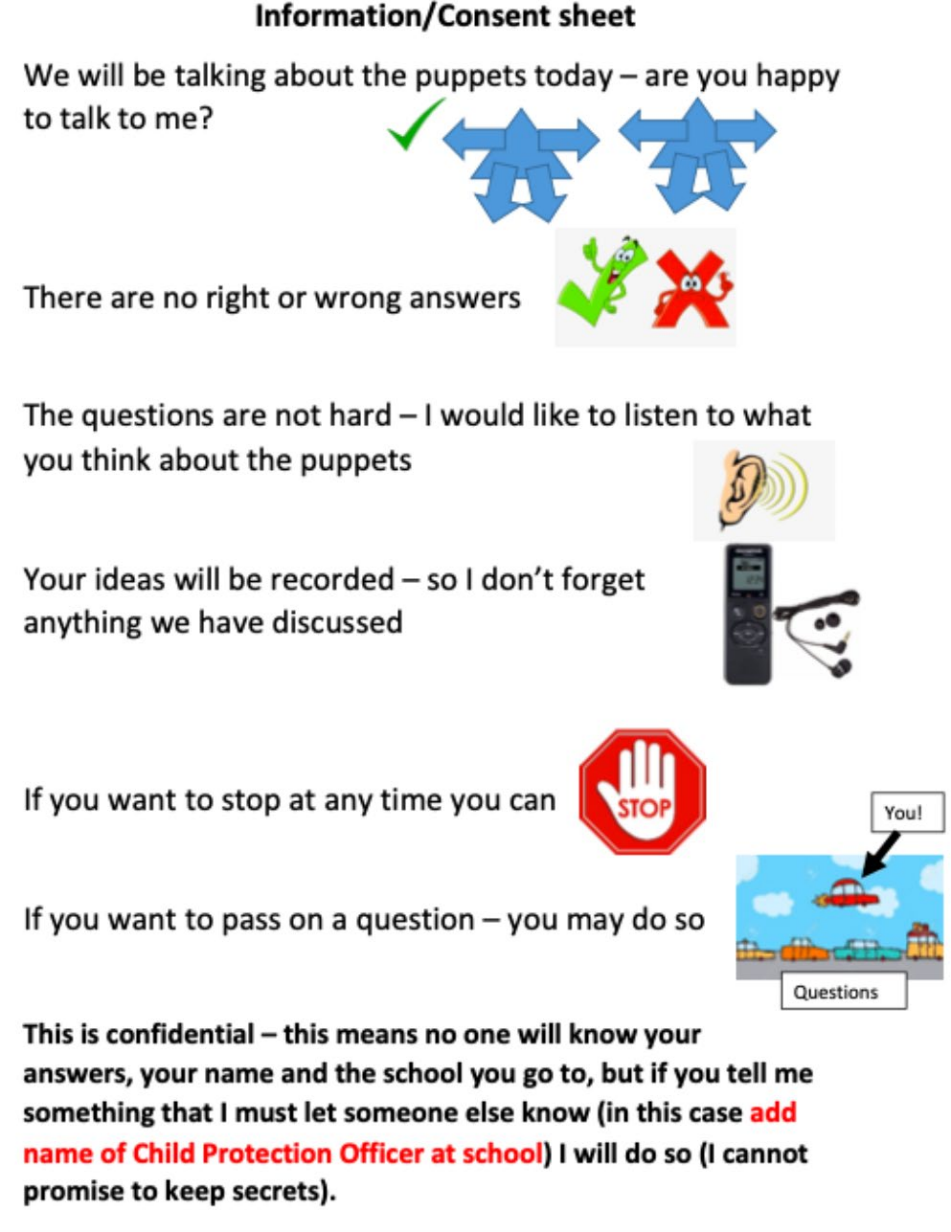
*Assent sheet shown and discussed with all children consenting to take part in the study*

A pictorial assent form, Fig. 1, was used at every point during the data collection, at pre-, post- and delayed post-intervention testing, allowing the children to stop or opt out from the research.

Pre, post- and delayed post-intervention testing activity was kept to a maximum of 15/20 minutes, which supported concentration skills and was age appropriate. EYFS children were asked to either point or say their ideas (which were recorded and transcribed).

In addition to this, if children showed distress e.g., by becoming visibly upset, uninterested or wanting to do something else – then they were withdrawn from the study. On two occasions this happened. First, in School 1 where a participant in EYFS began the study but lost interest after looking at the characters and the second participant, in School 2, in Year 2 looked visibly upset when asked to complete the questionnaire using an ipad. On both occasions it was ensured that children were okay, and they were supported in the classroom (after explaining to their teacher it was okay to stop).

## Reflexive Statement

It is likely that the researchers had some preconceived ideas about whether and why children may hold certain views about peers with learning and behaviour difficulties or about genetic screening, as a behavioural geneticist (KA) and a former teacher and Special Educational Needs Coordinator in primary schools (DF). However, the manner in which the older children’s viewpoints were derived (online questionnaire) reduced any influence that an adult may have. The risk was greater for the youngest children who provided their views verbally to the first author. To redress the imbalance of authority between the researcher and children, and to give children the confidence to provide their views (Lundy and McEvoy, 2017), three mitigations were used: firstly, taking on the character of ‘least adult’ (Mandell, 1991). Over several visits to the schools the first author (DF) became ‘a familiar figure’ to whom children appeared to feel comfortable and safe expressing their views (Mayall, 2008). Secondly, the use of vignettes depersonalised this sensitive topic (Schoenberg & Ravdal, 2000) and thirdly, by using puppets and providing children with developmentally appropriate methods of data collection we aimed to conduct research with rather than on children. However, it is important to acknowledge that the research was designed and administered by adults rather than being genuinely co-constructed with children (Facca et al., 2020). It is also important to acknowledge that the researchers, while aiming to be as objective as possible, are likely to have developed the vignette questions and interpreted the data through the lens of their experiences, biases and assumptions.

### Open Research and Pre-registration

The study was registered with the OSF to ensure the materials and analyses were fully transparent and could be replicated (Foster & Deardorff, 2017). See Open Science Framework (OSF) https://osf.io/n4dqp/ for pre-registration details and school implementation timetables and the intervention outline. The benefits of pre-registering the study included stipulating the hypotheses and analysis plan ahead of the data collection to stop hindsight bias when generating hypotheses, and confirmatory bias when testing hypotheses (Nuzzo, 2015).

## Measures

The children were asked to respond to four vignette-based questions, following a four session Philosophy 4 Children (P4C) intervention focused on learning and behaviour (P4C intervention). These sessions are not analysed here but represent the background to the current study. The series of 4 × 1-hour weekly P4C sessions focused positively on different learning difficulties, behaviour problems and conditions associated with them e.g. genetic conditions such as Down Syndrome and neurodivergent conditions such as autism. The questions and session context covered were as follows:

**Session 1 ‘What Makes Me, Me?’** What does my superhero label say about me?

The session stimulus was a book called ‘Don’t Call Me Special’ (Thomas, 2002) which focuses on addressing misconceptions about a wide range of conditions, using factual information and reflective questioning to challenge stereotyping. Children were asked to explore what they were good at and why they had chosen their superhero name (which was added to a lanyard and used throughout all P4C sessions). During the session the teacher introduced the idea that the super-hero name chosen might not be accepted by the group. Children were asked to suggest some reasons why this might happen, and if they could suggest ways they could ‘fix’ this super-hero so that it was ‘perfect’. The final reflective part of the session asked children to consider if they thought there could ever be a perfect super-hero and to reflect on the implications of being different.

**Session 2 ‘Differences’** What is it like to know someone who is different – does it matter if others think or behave differently?

The second session was developed to facilitate discussion around neurodivergence. The children were asked what is it like to know someone who is different and if it really mattered if others think or behave differently? The text chosen was a book called **‘**Isaac and His Awesome Asperger’s Superpowers’ (Dowling, 2016). Children in the session were encouraged to think about knowing someone who might have the same superpowers as Isaac. They were asked to also consider their own similarities to and differences from Isaac, and how they felt about them. The final reflection asked children to consider if their P4C discussion was likely to change what they thought or did in relation to children who are different to themselves.

**Session 3** ‘**Knowing and loving someone who is different’** What is it like to know and love someone who is different?

The third session used two stimuli. Reception and Year 2 were read a text called ‘ We’ll Paint the Octopus Red’ (Stuve-Bodeen, 1998). The book tells the story of a little girl who is expecting the arrival of a new baby brother or sister and imagines all the things they will do together. The story then explains that her little brother is born with Down Syndrome, a genetic disorder, and this makes her worry that they won’t be able to enjoy all the things she had imagined.

Year 5 were read a page from a different text called ‘Views from Our Shoes: Growing Up with a Brother or Sister with Special Needs’ (Meyer, 1997). This collection of children’s reflections gave some insight into the feelings of siblings who have a family member who differs from the non-disabled or neurotypical norm.

Although two different texts were used, the session was developed to facilitate discussion around what the children in the stories felt about their siblings.

**Session 4 ‘Differences from the inside or outside?’** Are babies different to each other when they are born or do they become different growing up?

Session 4 was developed to facilitate children’s philosophical dialogue in order to gain an understanding of their perceptions of the aetiology of individual differences, the subject of the current study. The question for the session was ‘Are babies different to each other when they are born, or do they become different growing up?’ The context for the session was an 8-minute BBC clip entitled ‘Same but Different, Life with Down Syndrome: Alexandra’s Story’, which shows the life of a little girl called Alexandra, who has Down Syndrome, told by her sisters. Alexandra is one of a set of triplet sisters. Her two sisters, Alicia and Felicia, look the same as each other (monozygotic). As Alexandra is different to her sisters in several ways the session aimed to get children thinking and discussing where the differences originated from.

Prior to the intervention children had been introduced to two grown up puppet characters - ungendered shape-based puppets called Zig (who finds learning difficult) and Zag (with behaviour problems) through a series of differentiated books (EYFS and Year 2) and comic strips (Year 5). Zig and Zag were not given any labels but participants were told about the behaviours they displayed within a school context.

During the P4C sessions, each of which lasted up to 1 hr, children were asked to discuss and debate the questions – with children directing dialogue and points of interest. Zig and Zag were present and visible at all sessions, as was the first author (DF) who audio-recorded and observed the sessions in silence. The class teachers, who facilitated each of the sessions, ensured that inquiries supported children to be Critical, Creative, Collaborative and Caring, the 4Cs, in their dialogue (SAPERE, 2021).

**Fig. 2:**
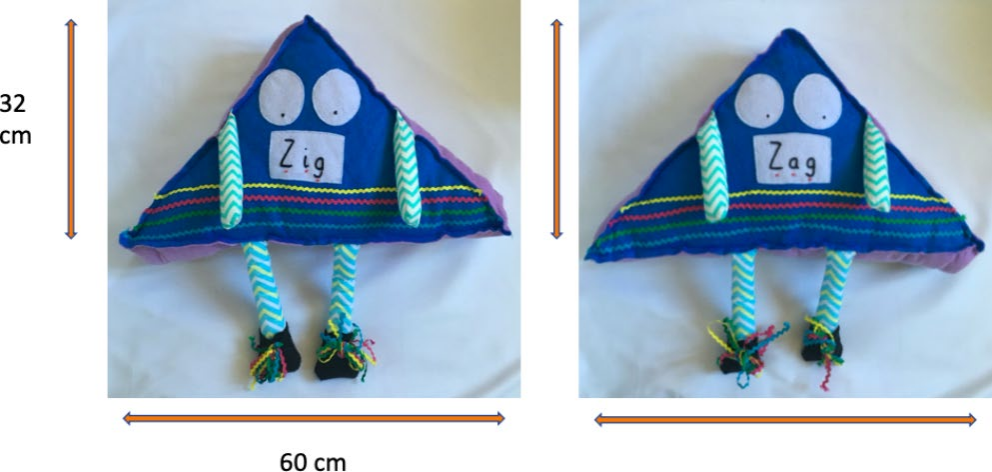
*Zig (Learning Difficulties) and Zag (Behaviour Difficulties) character measurements*

Later, during post-intervention data collection, the children were introduced to the idea of a new ‘baby’ shape called Zeggy, as part of the vignette questions that formed the data collection tool for the current study. Figure 2 shows the characteristics of the Zig and Zag puppets.

Children in Year 2 and 5 responded to the vignettes in writing, as part of an online questionnaire, while the first author worked verbally 1:1 with the youngest children, recording and transcribing their responses. The vignettes and questions were as follows:

- When Zig or Zag grows up it falls in love with another shape called Zog. They have a beautiful baby shape called Zeggy. They worry that baby Zeggy might find learning or behaving properly tricky like Zig/Zag. **Do you think they should be worried about this**?
- They hear that you can find out whether there is a good chance of baby Zeggy finding learning or behaviour tricky by going to the doctor for a test that doesn’t hurt a bit. **Do you think they should do this?**
- If you were Zig, Zag or Zog **would you test baby Zeggy?**
- What do you find tricky at school? If you’d had a test when you were a baby that showed you would find this tricky **do you think you, your family or your school might have been able to do anything about it?**

The vignettes used age-appropriate language and terminology. They did not specifically refer to genetics or genetic testing but highlighted that undertaking a test meant children could find out about how likely it was that baby Zeggy would grow up with learning or behaviour problems. They were sense-checked in a feasibility test with four children prior to being used.

## Coding and Analysis

An inductive, exploratory content analysis (Bengtsson, 2016) was chosen because the dataset was made up of a large number of relatively short statements from the children and was nested within a multi methods study (Teddlie & Tashakkori, 2009). Initially the full dataset was read several times and then broken down into the smallest meaning units possible without losing meaning, a process known as “decontextualization” (Bengtsson 2016, p. 11). The meaning units were then coded by labelling each with one or more codes. Coded data was then checked against the original data to check that all text with meaning had been coded i.e. recontextualised (Bengtsson, 2016). After recontextualising the data, codes were combined into categories. Coding was manifest rather than latent, focusing on what participants said rather than trying to identify hidden meanings. A codebook of 38 codes was developed and used to train a second researcher who independently coded 20% of the data in order to assess the reliability of the coding. Children (*n* = 128) reported a response to each of the four questions (*n* = 640 responses) and every fifth response (*n* = 128) was coded by the second researcher. We tested for intercoder reliability using Cohen’s Kappa and the results of this analysis are shown in a table which can be found on the project’s OSF page https://osf.io/n4dqp/page.

Cohen’s Kappa measures the level of agreement between raters, with 0 indicating no agreement and 1 indicating perfect agreement. Syed and Nelson (2015) suggest the following levels of agreement: k = 0.01–0.20 none to slight; 0.21–0.40 fair; 0.41– 0.60 moderate; 0.61–0.80 substantial; and 0.81–1.00 almost perfect agreement. In this study the initial average kappa = 0.75 agreement. We found almost perfect agreement from the outset for 16 out of the 26 codes, but only slight to fair agreement for the other 9 codes. Discrepancies were addressed by discussion between the two coders which led to some changes as shared understanding was achieved. After these changes, average kappa = 0.93 agreement, with near perfect agreement for 25 out of the 26 codes. There was still only slight agreement for Code 33 - Ld/bd viewed stereotypically (negatively), even after discussion, and so it was excluded from the analysis.

## Results and Discussion

Table 1 1 shows the six categories derived from content analysis to answer the research questions. The codes making up each category are listed, along with a brief description of the category. Some children chose a superhero name to use during the four P4C sessions and when responding to the vignettes, while others used initials. Their chosen names are presented throughout the results and discussion, and their words in Table 2.

**Table 1:** Frequencies for codes

Category	Code	Frequency
Worried	Not worried about learning or behaviour differences - you’ll get help	10
	Blame for learning/behaviour needs	3
	Worried about coping with learning or behaviour differences	8
	Worried about bullying	2
	Differences reduce your life chances	1
Origins	Behaviour is learned	11
	Behaviour can be self-controlled	1
	Viewed as vaccine/protection	13
	Genetic determinism	17
T Harmful	Doctors are careful	7
	No say - it’s unfair	4
	Testing dangerous - physically	45
	Testing dangerous - mentally	15
	Rude	1
T Helpful	Viewed as illness, needs curing	10
	Parents obliged to test	8
	Hospitals will help	1
	Allows for implementation of support	33
	Learning is key to getting on in life	6
	Testing is helpful	19
	Testing in itself will make life/learning/behaviour better	15
	Prefers low tech strategies after testing	35
How soon?	Find out when you are ready to know - wait until school	6
	Worry when we’re older and going to school	4
	Too Late	1
Point?	Loved anyway	14
	It’s not that bad!	24
	Testing is only for ill people	2
	Children might be different to parents	6
	Testing cannot predict behaviour	3
	You will learn anyway	3
	Tools or medicine might not be available	3
	What’s the point? Creates worry	3
	Schools cannot help you	41
	Child see pros and cons	7
-	Not Coded	28
-	Financial cost of testing	1
-	Don’t know/no response	205

**Table 2:** Categories derived, with associated codes

Category	Codes	Example quotation
Worried about being – and being seen as - different	Not worried - you’ll get help for learning and behaviour differences	‘Because people look after them’
	Blame for learning/behaviour needs	‘Because he might get told off… go in by their own with the baby… cos they don’t know what do because they are worried’
	Worried about coping with learning/ behaviour differences	‘Because they would be worried about Zeggy’s education and how he would act at school’
	Worried about bullying	‘Because someone might bully her at school’, ‘because she might get bullied at the park’
	Differences reduce life chances	‘Because if he can’t learn then he will never get anywhere in life’, ‘they’re worried he might do the things very wrong’
Beliefs about the Origins of Learning and Behaviour	Behaviour is learnt	‘Because he might make really good friends and they might help him behave properly and learn properly’, ‘If the baby was like zag they could do something better than zags parents and raise a better child’
	Behaviour can be self-controlled	‘You could be able to control everything and you don’t get any problems when you’re sad or angry’
	Viewed as vaccine/protection	‘Because he might get a bit poorly if he didn’t, so it keeps them healthy’, ‘Because they don’t won’t [sic] to go in the hospital’
	Genetic determinism	‘Yes, because the gens [sic] will go in the baby’
Testing is harmful	Doctors are careful	‘Cos er. he might be a little bit erm… it might be a little bit tickle… but that … when it tickles it makes me laugh! Cos it’s kinda good cos the doctor is really really careful to you!’
	No say - it’s unfair	‘Because she might not like it…. What the doctors going to do’, ‘No because they might already have signed in and they won’t let them cancel it’
	Testing dangerous - physically	‘It might be dangerous – what if they didn’t test it on someone else because it might damage her brain because you don’t know what it is’
	Testing dangerous - mentally	‘Because if they don’t like… everyday they will be hurt’,’ because it can impact their life up until birth because they would worry and be unsettled’
	Rude	‘Because it’s rude…. I don’t know’
Testing could help	Viewed as illness, needs curing	‘Because they want their baby to be helfy [sic]’
	Parents obliged to test	B’ecause [sic] they have to make their baby has to go there to listen’, ‘It is their decision’
	Hospitals will help	‘Yes, maybe make me in hospital’
	Allows for implementation of support	‘because it might help Zeggy’, ‘so you can help him’, ‘no because they should give him/her a chance and if he/she has trouble they should teach him/her’, ‘they could not make it go away but they can help you with your learning’, ‘because sometimes jeans [sic] pass on and sometimes they don’t’
	Learning is key to getting on in life	‘She needs to learn more and be smart enough because when she’s big she’s going to go to college and after college she might be having driving lessons’
	Testing is helpful	‘Because it is good for Zeggy and it does not hurt a bit’
	Testing in itself will make life/learning/behaviour better	‘It’ll help me learn and it will help me be clever!’, ‘so that actually make others happy by not being naughty any more…’
	Prefers low tech strategies after testing	‘Their mum and dad could help you if you can’t talk and walk and eat or sleep properly’, ‘my family could do something… maybe if they give him a fidget toy when he is learning that might help him a little and calm him down when he is behaving in bad w [sic]’, ‘some people have autism and the school give them ear defenders to cope with how loud it is and the school also bought and fitted a lift because there Is a boy in year six and he is disabled’
How soon is too soon for testing?	Find out when you are ready to know – wait until school	‘It wouldn’t understand and it would find out it was about when it was younger’, ‘Why take a test if you can find out on your own’
	Worry when we’re older and going to school	‘But if it was a new born I would get the bay [sic] tested as it is a baby and they shouldn’t be jumping to conclusions’,‘They shouldn’t hurt her and find out when she goes to school’
	Too Late	‘Because they’re not babies anymore’
What’s the point?	Loved anyway	‘Even if Zeggy grows up to have learning disabilities Zig/Zag and Zog won’t love him/her any less’, ‘I like the way he is and I love him’
	It’s not that bad!	‘Something more worrying might happen to other people’, ‘yes because they wanted him to be here’
	Testing is only for ill people	‘Because he’s not poorly’
	Children might be different to parents	‘Because i think that Zeggy will be different to Zig and zag’
	Testing cannot predict behaviour	‘Because they might grow up to be a good boy or a little girl. There is a chance that the test could be wrong and that just worries me more’
	You will learn anyway	‘Because the shape can learn and then it can go back to school because he will learn’, ‘Because she learns things’
	Tools or medicine might not be available	‘No, because you can’t do anything about it because they might not have the medicine to make them better’
	What’s the point? Creates worry	‘There is no point taking a test. All it is going to do is worry you. If you had a test and it told you you [sic] had learning disabilities you would be worried. Why bother putting a label on it when you can find that out for yourself’
	Schools cannot help you	‘Being loving… no because my school doesn’t do that… and I’ve started this school….’,’ Er… learning… it’s cos they can’t help you at school… it’s cos they can’t…’,’No because it will be tricky to learn’
	Child See Pros and Cons	‘because if he had the test he might get nightmares about the doctors if he once kneaded to go to the doctors for an emergency he might be scared to go and refuse to go But if he had it done you might get a benefit of knowing if he had learning difficulties and having him get extra help’

The frequencies for the agreed codes are shown in Table 3 and are discussed throughout each category apart from ‘don’t know’. This was a very frequent code (*n* = 205) used when children were unable to articulate or did not want to explain their reasons for choices made.

**Table 3:** Breakdown of category frequencies per year group (based on how often one or more codes within each category were applied to data from children in each year group)

Category	EYFS Frequency	Year 2 Frequency	Year 5 Frequency	Total Frequency	
**Worried**	22	15	16	53	
**Origins**	8	18	16	42	
**Testing Harmful**	32	27	13	72	
**Testing Helpful**	32	44	51	127	
**How soon?**	2	3	6	11	
**Point?**	51	28	27	106	
**Don’t Know/no response**	90	84	31	205	

The frequencies for each category have been broken down into year group responses in Table 3. The frequency of the Don’t know/no response code suggests that EYFS children responded with don’t know or made no response (*n* = 90) slightly more readily than Year 2 children (*n* = 84) and noticeably more readily than Year 5 children (*n* = 31). This could arguably be because children were unable to respond to the questions due to their language or comprehension ability, and therefore gave ‘don’t know’ answers, but also because they may be applying their ‘right not to speak, which must be respected’ (Kirby, 2020, p. 821), particularly in the case of EYFS children who were interviewed on a 1–1 basis. For these children the dynamics of an adult-child power relationship could have led them to not want to offend or disappoint the adult with their answers (Kirby, 2020). It could also be the case that the questions evoked a negative feeling – which the children may not have wanted to address (Courchesne et al., 2021). However, it is important to remember that children were presented with a pictorial assent sheet which explained that they could stop at any time; move on to another question; or withdraw from the research with no consequences. Our novel method may also have contributed to the number of children making a choice not to answer questions (by leaving written answers blank in the case of Year 2 and Year 5) (Courchesne et al., 2021). A breakdown of ‘don’t know’ responses by question highlighted that EYFS children may have found some of the questions posed to them particularly difficult to answer or perhaps they didn’t want to answer questions about their own views. For example, the most ‘don’t know’ responses from EYFS children were in relation to a question which asked them to consider whether, if a child found something tricky, their family, their school, or they themselves could do anything about it. Year 2 children gave 29 ‘don’t know’ responses to a question asking if Zeggy’s parents should feel worried about Zeggy potentially being born with learning difficulties or behaviour problems - potentially indicating that developing a perspective on this was difficult for them. Year 5 children indicated a smaller number of ‘don’t know’ responses to the question about whether the parents should test Zeggy for learning or behaviour problems, and although a smaller number of children provided this response, it potentially indicates that some children were not able to put themselves into the parents’ shoes.

## Category 1: Worried About Being – and Being Seen as – Different

This category is about children’s perceptions of being - or being seen as – different. Table 3 indicates that children aged 4–5 years provided slightly more responses regarding being worried (*n* = 22) than children aged 6–7 years and 9–10 years (*n* = 15 and *n* = 16 respectively) although the difference was not particularly marked.

Children suggested Zag and Zog, as parents, should be worried about baby Zeggy being born with behaviour problems because ‘sometimes they might be a bit naughty’ (CB) and so they might be ‘worried he might do the things very wrong’ (AH). They also suggested being born with behaviour differences would affect how Zeggy might react to situations such as becoming ‘so horrible and nasty to each other…. because that’s what me and my sister do when we are in trouble’ (LB). They worried about outbursts of aggressive behaviour, questioning ‘what if the baby smacks or punches somebody?’ (TTU). One child suggested someone with behaviour problems may not be trustworthy ‘because they could be fibbing’ (Galaxy Girl). Some children indicated that children with behaviour problems ‘aren’t really good friends’ (PC) and ‘they are really annoying!’ (FB). In summary, they expressed a wide range of negative views about behaviour problems.

Children also voiced concern about being born with learning difficulties ‘because they might not know how to do a lot of stuff’ (Batman) and ‘cos they don’t know what to do” (FG). Children feared having learning or behaviour difficulties would affect life and self-help skills ‘because they might need help - it might not know how to get dressed or brush his teeth’ (Sooper Happie). Worry was also expressed by the children about having specific challenges, for example being unable to read and write well, and showing impatience and expressing themselves physically. Children perceived that children with these issues were more likely to be intimidated and bullied, explaining that Zeggy would get **‘**bullied at the park’ (Brave Rose) and that ‘someone might bully her at school’ (Sparkly Reenie).

One child was concerned with being blamed for being born with learning or behaviour difficulties ‘because he might get told off’ (HR). Fundamentally, there was a perception it might be hard to be a child with learning difficulties and it might be hard to be around a child with behaviour problems.

However, it was felt by some children that even if you are different and need extra help it could be worrying but it would be okay because you could get help:

I think my family would be able to help because they could help you by treating you. And school because they could treat you good as well and give you toys to fiddle with and help you concentrate and learn more stuff for when you grow up. (Secret Spy)

Overall, the data in this category indicated a substantial proportion of children in the sample expressed worry and concern about being – or being seen – as different; and had mainly negative views of what life is like for children with learning or behaviour difficulties. Negative perceptions focused mainly on difficulties in being able to form friendships, the perceptions held by teachers, potential expectancy effects (Shifrer, 2013), the potential for being bullied and difficulties with learning and everyday tasks.

## Category 2: Beliefs About the Origins of Learning and Behaviour

This category focuses on the children’s perceptions of the causes of learning and behaviour difficulties, both genetic and environmental, although not necessarily labelled as such by the children. We found some children believe learning and behaviour difficulties will inevitably be transmitted from parent to child, while others believe they can be altered by parents, family and peers. Overall, there were 42 responses related to the causes of learning and behaviour difficulties. There were 8 responses from 4 to 5 year olds, 18 from 6 to 7 year olds and 16 from 9 to 10 year olds, suggesting that 4–5 year olds may not have been quite ready to engage in this way.

The 9–10 year old children seemed particularly convinced of the inevitability of Zeggy having learning or behaviour difficulties because Zig/Zag did. This suggests a belief in genetic determinism, as children explained it would definitely be the case ‘because there [sic] genes are the same’ (OG), ‘the gens [sic] will go in the baby’ (Dance Drama) and a more moderate view that ‘Zeggy might have problems because their parents did’ (Awesome Artist). Secret Spy explained further, ‘Zeggy could act like Zag and be very nasty or he could be like Zig and can’t read and needs lots of help’. Children also made comparisons between Zig, Zag and Zeggy’s learning as ‘Zeggy might find it tricky because Zig and Zag did’ (Super Writer).

Children suggested, after reading the resources about Zig (learning difficulties) and Zag’s (behaviour difficulties) characteristics, that they would expect to see the same learning and behaviour difficulties in any children ‘because Zig/Zag are clumsy and talk like babys [sic]. So Zeggy will do that to [sic]’ (EB). Children saw this as inevitable and made the case that Zeggy would not be able to learn and that this has a long-term impact ‘if he can’t learn then he will never get anywhere in life’ (Mathematical Man). They expressed negative beliefs and assumptions about learning and behaviour difficulties as well as genetically deterministic views.

However, some children also made environmentally deterministic assumptions and blamed parents for poor behaviour in their children. For example, Mrs Brave suggested Zeggy would behave badly ‘because they don’t behave either’, and parents should be worried ‘because they don’t want it to be like them’ (Bob the Brilliant Braille Boy). As TR explains: ‘Sometimes babies learn from their parents and do it themselves and that can be worrying’. This suggests some children believed learning and behaviour difficulties are learned from parents. In this vein, CB2 indicated good parenting could change behaviour ‘if the baby was like Zag they could do something better than zags [sic] parents and raise a better child’. HH explains that changes in behaviour could be made by friends and school helping:

you shouldn’t worry because he might make really good friends and they might help him behave properly and learn properly and you could help him to [sic], or he might have a really amazing teacher that will help him so just don’t worry. If he was like Zag, then he might be able to help himself and behave properly.

Learning and behaviour difficulties were also likened by some children to illnesses which could be cured. FM suggested ‘he’s poorly…. Like my leg!’. Sparkly Reenie explained: ‘just in case there’s a problem when she gets pain and sometimes she needs a test to see if her blood works properly’ and AT said ‘they might be able to save it!’

It was also noteworthy that there was some evidence of a deterministic role for gender, with a striking number of children making the assumption that Zeggy was male. The majority of responses referred to Zeggy as male (EYFS, 20 responses, Year 2 30 responses, and Year 5 31 responses). Zeggy was only described as female in one response from children in EYFS, 10 in Year 2 and 0 in Year 5. An example of this is when asked about testing Zeggy for learning difficulties or behaviour problems, Emma Cat Loo says:

I think they should because they can find out if he Autistic or ADHD or not and they can not worry and they can get a support (Emma Cat Loo)

However, the character Zeggy was introduced to the children as an ‘it’ triangle shape – just as the characters Zig and Zag had been. This could be an indication of the children looking for hidden and underlying meaning, categorising the characters who struggled with either learning or behaviour as more likely to be male (Gelman, 2004).

In summary, some participants believed that genes are destiny, and a parent who struggles will inevitably have a child who struggles, while others adopted a more blank slatist position, believing child development is governed by environmental factors such as parenting, friendships and school and therefore positive environments were all that was needed to address any ‘negative’ learning or behaviour difficulties. There was clear evidence of essentialist thinking in both directions, including around the role of sex or gender, indicating that children formed ‘causal explanations’ (Gelman, 2004, p. 404) for differences.

## Category 3: Testing is Harmful

This category is about children’s negative perceptions of testing for risk of learning or behaviour difficulties, although some children approached it by thinking about their experiences of medical and diagnostic testing in general. For example, they related it to visiting their own doctor or having tests for other medical reasons. Overall, 73 responses related to whether participants perceived testing to be harmful. The 4–5 and 6–7 year olds worried the most about Zeggy being hurt by the test (32 and 27 codes applied respectively) with 9–10 year olds worrying less about this (13 responses), which suggests some possible changes in understanding and/or priorities over time. Table 1 shows that the two most frequent codes in this category were children believe testing to be harmful physically (*n* = 45) and mentally (*n* = 15).

When asked whether Zig/Zag and Zog should have Zeggy tested children expressed concern that the testing process itself would be harmful, commenting: ‘I don’t want any babies to cry’, (PP). As one child put it: ‘I’ve been to the doctors… I just had needles, but it did hurt’ (JH). Children were also concerned about whether testing could physically alter someone. Dancing Flexibility Superstar wondered if ‘it might damage her brain because you don’t know what it is’ and Suggar Rush questioned the safety of testing ‘it might be dangerous – what if they didn’t test it on someone else?’. Tianise explained a longer-term worry:

If he got it I think that I would cry because I don’t want him to change I like the way he his [sic] and won’t want to go there ever because sometimes things change and his face might change and not be the same as it was when he didn’t have the test.

Their concerns about physically hurt during testing were not alleviated by the introduction of the scenario stating that genetic testing ‘did not hurt a bit’.

As well as expressing concern for Zeggy, CB2 also suggested knowing about learning and behaviour difficulties through pre-natal testing could have long term mental health effects on the parents, Zig/Zag and Zog too, as ‘it can affect their life up until birth because they would worry and be unsettled’.

Some children considered how testing might infringe their rights, with JD indicating that they thought testing was ‘rude’ and AH showing concern for Zeggy’s rights, suggesting unfairness as Zeggy had no say in the situation ‘cos she might not like it…. what the doctor’s going to do’. Galaxy Girl highlighted the unfairness of how children’s consent for testing is not sought and the lack of autonomy to withdraw ‘because they might have signed in and they won’t let them cancel it’.

SL showed concern about confidentiality and everybody else finding out before them, and withholding this information about themselves ‘Cos what if I was so little and went to the doctors before someone told me? And they didn’t tell me so…so…so… I don’t think they should do it…’ This was also reiterated by Elme who explained that decisions made about testing should be up to the parents of the child being tested and not anyone else. Note that Elme, like several other children, conflated learning and behaviour difficulties with neurodivergent conditions such as autism.

I don’t think they should get a test done as they will know what will happen in his life so it wouldn’t be a surprise … but I guess it is up to its parent not up to other people as they might have autism and it’s better to be safe than sorry but also I think they should keep an eye out for the key features of Autism because you never know but If it’s a new born I think they should wait a while before jumping to conclusion on if he will be bad at school or naughty but as I said in a way it’s not up to me it’s up to the parent(s) of the child.

Some children however suggested because of the carefulness of doctors testing would be okay, which seems to suggest that doctors make the testing less dangerous and that it would be similar to undertaking routine health checks ‘because I like being [sic] to the dentist - it’s the same’ (IR).

In talking about testing children expressed a desire for baby Zeggy not to be hurt, either physically or emotionally. They were also able to engage in some sophisticated thinking around the right to know, and the right of the child not to know, and about the risks inherent in trying to predict the future for both parents and children. While Category 1 identified some very negative and deterministic views about learning and behaviour difficulties, children were still concerned about testing for them - primarily because of concern for Zeggy.

## Category 4: Testing Could Help

This category is about children’s attitudes towards testing and if they perceive it to be helpful. Some children argued testing would be helpful as it would allow for implementation of extra support. Overall, 127 responses expressed the view that testing could help with identifying learning and behaviour difficulties, seeing this as a good thing. This suggests some children who pointed out that testing could be harmful also acknowledged ways in which it could be helpful. Indeed, 9 children from EYFS, 8 children from Year 2, and 6 children from Year 5 held both ideas simultaneously. This data indicated that holding these dual perspectives were aligned well with those of a series of expert panels set up to explore a similar question (Asbury et al., 2021). The number of children expressing this viewpoint increased somewhat with age, with 32 responses from 4 to 5 year olds, 44 responses from 6 to 7 year olds and 51 responses from 9 to 10 year olds, the opposite pattern to that observed in Category 3. Table 1 frequencies also noted that children perceived that testing would lead to support (*n* = 33), testing would be helpful (*n* = 19) and children indicated a preference for low tech strategies after testing (*n* = 35).

AO explained that knowing about learning difficulties would help because it could inform intervention: ‘people could understand how to help him’. By identifying strengths and weaknesses which could as MS put it ‘tell them what is the matter with him’ and would allow children with learning and behaviour difficulties ‘to see if they can learn a bit more’ (IJ).

It was felt by some that testing would be helpful for parents and school to ‘get prepared’ or ‘have a heads up’ (OG). The Acro Star suggested testing ‘should give him/her a chance and if he/she has trouble they should teach him/her’ and in turn reduce fear ‘they could tell us don’t worry then you wouldn’t be scared’ (EB1).

Getting tested was important for Super Turtle as they questioned whether children with learning and behaviour difficulties had developed these ‘because they have germs’ and Super Ellie explained testing might make learning and behaviour difficulties ‘go away’. Super Turtle‘s point may also highlight a basic understanding that something inside of you may be affecting how you develop, which testing for will help. Hulk Smash suggested not going to the doctors would be detrimental ‘because if you don’t go to the doctors it might not go’, and this was reiterated by OH and Math Man. This betrayed a misunderstanding, equivalent to the belief that testing would cause the problem, that testing would cure it. Indeed, some of the children explicitly stated the misconception that a predictive test for learning and behaviour difficulties would act as a cure and described how others would benefit, as well as Zeggy, saying it would ‘actually make others happy by not being naughty any more’ (LB).

Children put themselves into others’ shoes by explaining that being tested for learning or behaviour difficulties could enable a variety of low-tech strategies and support to be provided to them. For example: small, stepped approaches in learning: ‘I think school would help with lessons by explaining what we are doing a bit more and possibly step by step but could mainly help with that’ (MM). The provision of resources would help as ‘they might be able to get stress toys for him and things to stop him getting distracted and get him special needs stuff’ (Emma Cat Loo), and by offering technological support ‘Sometimes if you’re struggling to do English right she might need to go on a computer’ (Sparkly Reenie). TTU used personal experience to explain how medical intervention could provide support ‘well I know that a doctor can help me I am getting medicine to concentrate more. I’m get [sic]medicine to concentrate’.

Elme indicated disclosure of difficulties after testing may help with managing others’ perceptions of learning and behaviour difficulties:

I think the school would help as I have seen they have helped with over [sic] people and the teacher at home I think your family will help you no [sic] stop as they are there for you when you are down but some friends not be able to as they might not know and you might upset them a lot but you always need to warn your friends. Also some family you haven’t seen for a while might mess with you and you might do something wrong so also warn them I would always warn my family.

CB explained testing ‘would be okay to have it mmm yeah - It’ll help me learn and it will help me be clever!’ This viewpoint may reflect a misconception that testing would enhance their abilities.

Dance Drama, when asked what they themselves found difficult, and if there was a test available could anyone do anything about it, explained that ‘they could not make it go away but they can help you with your learning’. Baller however, had mixed feelings and could see both the positives and negatives of being tested for learning or behaviour difficulties

because if he had the test he might get nightmares about the doctors if he once kneaded [sic] to go to the doctors for an emergency he might be scared to go and refuse to go. but if he had it done you might get a benefit of knowing if he had learning difficulties and having him get extra help..

In summary, some children perceived testing to be helpful, even some of those who also saw it as potentially harmful. In this category children explained testing would be helpful as this would allow for the identification of strengths and weaknesses and for the implementation of support.

## Category 5: How Soon is Too Soon for Testing?

This category is about what children perceived to be the optimal timing for DNA screening for risk of learning and behaviour difficulties. Very few children spontaneously considered this issue (two responses from 4 to 5 year olds, three responses from 6 to 7 year olds and six responses from 9 to 10 year olds) but it is interesting to consider their views because it sheds some preliminary light on when children believe they have capacity to understand, and the age and timeframe that is appropriate.

For some, the idea of testing young children was a difficult thought as babies don’t know why they are being tested ‘because babies can’t erm… they don’t know…’ (AB). In simple ways these children spontaneously considered the rights of baby Zeggy. Children put themselves into the shoes of a baby and explained they would be too young, ‘he’s only little’ (EB1).

Waiting until you’re older to test was a view shared by AM1 who explained parents ‘can worry about that when their baby is older’. This viewpoint was also expressed by FH explaining ‘they shouldn’t hurt her and find out when she goes to school’. IH made the point it is too soon to test until other things have been tried. And when difficulties are identified a graduated approach of low-tech strategies might help someone with learning or behaviour difficulties.

I don’t know if I would because if he did have trouble learning then I would try and tech [sic] the baby first then if that doesn’t work then see if the teachers could maybe help and if that doesn’t work then I may take it to the doctors.

Conversely MD indicated testing when children were older would not be helpful as it would be too late ‘because they’re not babies anymore’, perhaps indicating a preference for early intervention.

In summary, this category indicates most of the children who considered the timing of predictive testing - a minority - felt that children should wait until reaching school age to test for learning or behaviour difficulties because by that point children would have more understanding of what was happening and why. However, one voice indicated testing when the child was at school may be too late.

## Category 6: What’s the Point?

This category is about children questioning whether testing can predict learning and behaviour difficulties in any useful way, and whether there would be intervention, medical or therapeutic, available after testing. Table 1 shows that the most frequent codes included: having learning or behaviour difficulties is not that bad (*n* = 24), schools can’t help anyway (*n* = 41) and the child would be loved anyway (*n* = 14). This category highlights that some children feel that testing is unnecessary as children will learn anyway, testing is only for ill people, and testing for learning or behaviour difficulties would create worry and distress.

I Don’t Know got straight to the point explaining

There is no point taking a test. All it is going to do is worry you. If you had a test and it told you you had learning disabilities you would be worried. Why bother putting a label on it when you can find that out for yourself. There is a chance that the test could be wrong and that just worries me more. Why take a test if you can find out on your own?.

OH agreed there was no point in testing ‘because you can’t do anything about it because they might not have the medicine to make them better’, recognising that findings from screening need to be clinically or educationally actionable to be justified.

Testing for some would create heightened worry about having learning and behaviour difficulties as explained by Sooper Happie ‘because you might have been born with it… and you may be it’s too worse to find out’.

One child commented that testing was only for ill people, explaining about Zeggy ‘he’s not poorly’ (HAB), suggesting learning and behaviour difficulties were not seen as illnesses by all, and so it would be inappropriate to test for them. Super Sports also made the point that perhaps we should not be thinking about such difficulties in a deterministic way but should wait to see what happened and judge the child on their own merits: ‘I think that Zeggy will be different to Zig and Zag’ (IB).

ES also commented that Zeggy may resemble one parent more than the other and testing may not be useful as other factors might impact on learning and behaviour ‘because they have quite different personalities so it will depend on which one Zeggy will be more like’.

DQA suggested learning and behaviour difficulties ‘comes and goes’, suggesting a view that learning and behaviour change was dependent on environmental factors and testing may therefore not accurately detect difficulties.

Some children, however, expressed uncertainty about the benefits of testing ‘because I don’t know if it’s a good thing to do or a bad thing’. Captain Helpful explained the difficulty in predicting behaviour ‘because I don’t know if he’s going to do bad or good behaviour’ and TR thought learning and behaviour difficulties were nothing to be concerned about ‘because something more worrying might happen to other people’. SW explained testing would be dependent on whether or not they would be having a baby and if parents thought there may be something wrong ‘because if they don’t think there is going to be nothing wrong with the baby then no, if there is something wrong with the baby then yes.’ It is interesting to note the children’s use of terms such as ‘wrong’ and ‘bad’, particularly in describing behaviour problems.

In spite of the very deterministic thinking evident in Categories 1 and 2 not all of the participants thought this way. For example, MM explains that having learning difficulties is nothing to worry about ‘even if that is true it will not be bad, there is only a possibility of this happening and if it would happen then it will be alright and the child will have possibly gotten extra help while learning anyway!!’. One child explained from personal experience that in time, and development, children with learning or behaviour difficulties would be okay explaining ‘I was worried before my sister was grown. She would not be able to do anything! But by the time she growed up she could do lots of things…’ (SL).

Being different, some children thought, would not be easy but felt support would be offered to provide a break for parents whenever needed ‘Cos they could always call a baby sitter…. That looks after them at their house… anytime!’ (RT).

SS suggested what was the point in testing because Zeggy would be loved anyway ‘because he’s good as he is’. I Don’t Know argued that ‘even if Zeggy grows up to have learning disabilities Zig/Zag and Zog won’t love him/her any less. They can get Zeggy the help he/she needs’.

Super K however suggests testing would not reveal anything useful as ‘I think that every baby is different in their own special way and I think that every baby has a different personality and like different things and different places and different food’. When asked whether, after testing for learning or behaviour difficulties, anyone could help with aspects of behaviour, FM suggested schools could not help him with being loving ‘because my school doesn’t do that… and I’ve started this school’.

In summary the data indicates some children were sceptical about the point of screening for risk of learning and behaviour difficulties, explaining there’s not much point because the information won’t be reliable or useful. In their way they identified the issue that we should not test for problems that we do not have clear solutions for, that is, that tests need to be clinically or educationally actionable to have value.

## Conclusion and Limitations

These findings show that even very young children, the youngest participants were just four years old, are capable of contributing to public debate in this area, especially when supported by an intervention which promotes discussion and provides a platform (Wall, 2017) for children to communicate their opinions. In light of this, responsible research into the future use of DNA data in education should seek children’s views and also explore ways of enhancing children’s genetic literacy.

It is noteworthy that the children in this study held negative deterministic perceptions of life with special educational needs and showed a tendency towards overly simplistic explanations for those needs. Developmental psychology literature suggests that this tendency fits with the development of psychological essentialism in young children (Gelman, 2004), and their growing preference for homophily by the age of four (Schwab, 2019). Further understanding the aetiology of these developing capabilities and perceptions represents a research priority for both genetic researchers and inclusive education researchers. It would also be a positive step to develop innovative and appropriate science communication approaches for young children regarding determinism and difference. This is important because we know that children make assumptions about others without being taught about categorization (Gelman, 1998) and it is possible that age-appropriate education can be used to counter any harmful effects of that, such as ‘othering’ those who are different.

A possible limitation or source of bias might be in the formulation of question 2. It is possible that in explaining ‘*that there is ****a good chance ****that baby Zeggy finds learning or behaviour tricky and that you ****can find out ****by going to the doctor for a test’* we could have inadvertently nudged some children towards expressing genetically deterministic views. However, there was no sign in the data that children responded to question 2 with more deterministic views than they did to the other three questions. We also acknowledge that vignettes posed questions which could be perceived as using the ‘medical model’ terminology (Pellicano & den Houting, 2022) that is widespread in schools (Alderson, 2018). It was not intended to lead the children or develop further negative embedded meanings but merely to pose questions to the children using terminology that they recognised and understood. Formulating questions for future studies on this topic, to reduce bias, could mitigate risks and possibility of harm, particularly for marginalized communities including children, by co-producing research materials, and indeed questions, with them (Liddiard et al., 2019). For example, the formulation of question two of the vignette, the test “doesn’t hurt a bit” was not intended to give the children the impression that such testing is free of risks and harm. It was simply intended to convey to the children that it would not involve physical discomfort. This formulation was based on the researchers’ experiences of working with young children. However, on reflection, we can see that it is possible that some children might interpret it as meaning it would not cause harm. While there was no evidence of this interpretation in the data it is important to consider that misinterpretation is possible and to bear this in mind in developing these kinds of materials.

This study goes beyond asking for children’s perceptions of DNA screening. It highlights the need for further support and development of children’s understanding and acceptance of diversity and the need for opportunities to develop and maintain friendships (Carter & Nutbrown, 2016). This could ultimately support genuinely ‘inclusive classroom communities’ (Black-Hawkins et al., 2021, p. 13). This discussion is highly relevant to educational genetics wherein issues of stigma and self-stigma, and also expectancy effects (Shifrer, 2013), need to be taken seriously (Asbury et al., 2022).

In order to support pupil success in education and the wider world, we need to work ‘with’ children as opposed to ‘on’ them (Dockett & Perry, 2011, p. 231). Engaging and listening to children’s perceptions of the idea of DNA screening for risk of learning or behaviour difficulties requires creative approaches and a focus on how children develop their understanding of complex topics such as diversity and genomic prediction. This could potentially be supported by high quality science communication within education that is accessible from the first days of primary school (Gus, 2000; Litvack et al. 2011; Cairns & McClatchey, 2013; Beckett, 2014; Armstrong et al. 2017; Black-Hawkins et al. 2021; Asbury et al., 2022). We argue that it is important, and responsible, to include children, even very young children, in consultations about the future of DNA data in the context of education.

## Data Availability

The datasets generated during and/or analysed during the current study are available from the corresponding author on reasonable request.
